# Age-related differences in the loss and recovery of serial sarcomere number following disuse atrophy in rats

**DOI:** 10.1186/s13395-024-00351-5

**Published:** 2024-08-02

**Authors:** Avery Hinks, Geoffrey A. Power

**Affiliations:** Department of Human Health and Nutritional Sciences, College of Biological Sciences, University of Guelph, 50 Stone Road East, Guelph, ON Canada

**Keywords:** Muscle architecture, Force-length relationship, Sarcomere length, Fascicle length, Pennation angle, Muscle thickness

## Abstract

**Background:**

Older adults exhibit a slower recovery of muscle mass following disuse atrophy than young adults. At a smaller scale, muscle fibre cross-sectional area (i.e., sarcomeres in parallel) exhibits this same pattern. Less is known, however, about age-related differences in the recovery of muscle fibre length, driven by increases in serial sarcomere number (SSN), following disuse. The purpose of this study was to investigate age-related differences in SSN adaptations and muscle mechanical function during and following muscle immobilization. We hypothesized that older adult rats would experience a similar magnitude of SSN loss during immobilization, however, take longer to recover SSN than young following cast removal, which would limit the recovery of muscle mechanical function.

**Methods:**

We casted the plantar flexors of young (8 months) and old (32 months) male rats in a shortened position for 2 weeks, and assessed recovery during 4 weeks of voluntary ambulation. Following sacrifice, legs were fixed in formalin for measurement of soleus SSN and physiological cross-sectional area (PCSA) with the un-casted soleus acting as a control. Ultrasonographic measurements of pennation angle (PA) and muscle thickness (MT) were conducted weekly. In-vivo active and passive torque-angle relationships were constructed pre-cast, post-cast, and following 4 weeks of recovery.

**Results:**

From pre- to post-cast, young and older adult rats experienced similar decreases in SSN (–20%, *P* < 0.001), muscle wet weight (–25%, *P* < 0.001), MT (–30%), PA (–15%, *P* < 0.001), and maximum isometric torque (–40%, *P* < 0.001), but there was a greater increase in passive torque in older (+ 180%, *P* < 0.001) compared to young adult rats (+ 68%, *P* = 0.006). Following cast removal, young exhibited quicker recovery of SSN and MT than old, but SSN recovered sooner than PA and MT in both young and old. PCSA nearly recovered and active torque fully recovered in young adult rats, whereas in older adult rats these remained unrecovered at ∼ 75%.

**Conclusions:**

This study showed that older adult rats retain a better ability to recover longitudinal compared to parallel muscle morphology following cast removal, making SSN a highly adaptable target for improving muscle function in elderly populations early on during rehabilitation.

**Supplementary Information:**

The online version contains supplementary material available at 10.1186/s13395-024-00351-5.

## Introduction

Natural adult aging is associated with a decline in muscle mass, occurring at a rate of at least 0.5% per year in humans after age 60 [1–5]. This loss of muscle mass is accounted for not only by losses of muscle fibre number and cross-sectional area [2, 5], but also by a reduction in muscle fascicle length (FL) [6–8]. Studies on animals have shown this age-related reduction in FL is driven by the loss of sarcomeres aligned in series [9–11]. A muscle’s serial sarcomere number (SSN) is tightly coupled with its mechanical performance including force production throughout the range of motion and resting passive tension [12–14]. Aging is accompanied by a reduced capacity to generate active force and an increase in resting passive tension, and the loss of SSN contributes to those mechanical impairments [5, 11, 15–17]. Loss of SSN and strength, and increased resting passive tension are also observed following periods of immobilization with the muscle casted in a shortened position [18–21]. These negative alterations to muscle morphology and function are important to consider as older adult humans are prone to falls and illnesses, making periods of cast-immobilization or bed rest common [22, 23]. These negative effects of immobilization could be additive on top of aging, worsening their trajectory towards loss of independence.

To mitigate or reverse the loss of SSN in both aging and immobilization, interventions promoting an increase in SSN have been proposed. Studies on young adult rodents have investigated the ability for serial sarcomerogenesis through a period of simply voluntary ambulation following immobilization-induced SSN loss. SSN recovered within 3–4 weeks following cast removal [24–26] likely due to the considerable stretch stimulus that ambulation imposed on muscles after being immobilized in a shortened position. This stimulus for serial sarcomerogenesis may, however, be limited in older adult rats because a stiffer ECM limits their range of motion and fascicle stretch during walking [27]. Furthermore, a decline in habitual physical activity likely contributes to the age-related loss of functional capacity in humans [28–30]. Similarly, older adult rats exhibit lower voluntary physical activity than young adult rats [31], which could further limit the stimulus for re-growth during voluntary ambulation following disuse atrophy.

Previous studies have provided important insight into age-related differences in recovery following disuse atrophy. In humans, Suetta et al. [32] observed a smaller loss of quadriceps muscle volume in older compared to young adult men following 2 weeks of immobilization, however, after 4 weeks of retraining, muscle volume recovered less in older than young men. In the same cohort, Suetta et al. [33] observed no recovery of vastus lateralis single fibre cross-sectional area (CSA) in older adult men following 4 weeks of retraining, but full recovery in young adult men. A similar blunting of muscle mass recovery following 4 weeks of immobilization was observed in older adult rats [34]. While these previous studies provided understandings of age-related differences in the recovery of whole-muscle mass and parallel muscle morphology (i.e., CSA) following immobilization, it is unclear whether there is also a blunted ability to recover SSN in old age, which is the driving mechanism of longitudinal muscle growth.

Comparing young adult (8 months) and older adult (32 months) Fisher 344/Brown Norway rats, the present study aimed to investigate: (1) age-related differences in the loss of soleus SSN and plantar flexor mechanical function during 2 weeks of casting in a shortened position; and (2) age-related differences in the recovery of soleus SSN and mechanical function during 4 weeks of voluntary ambulation following cast removal. We hypothesized that older adult rats would exhibit a smaller magnitude of SSN loss than young adult rats following immobilization, however, would take longer to recover their SSN, contributing to impaired mechanical function and blunted longitudinal muscle growth.

## Methods

### Animals

**Fig. 1:**
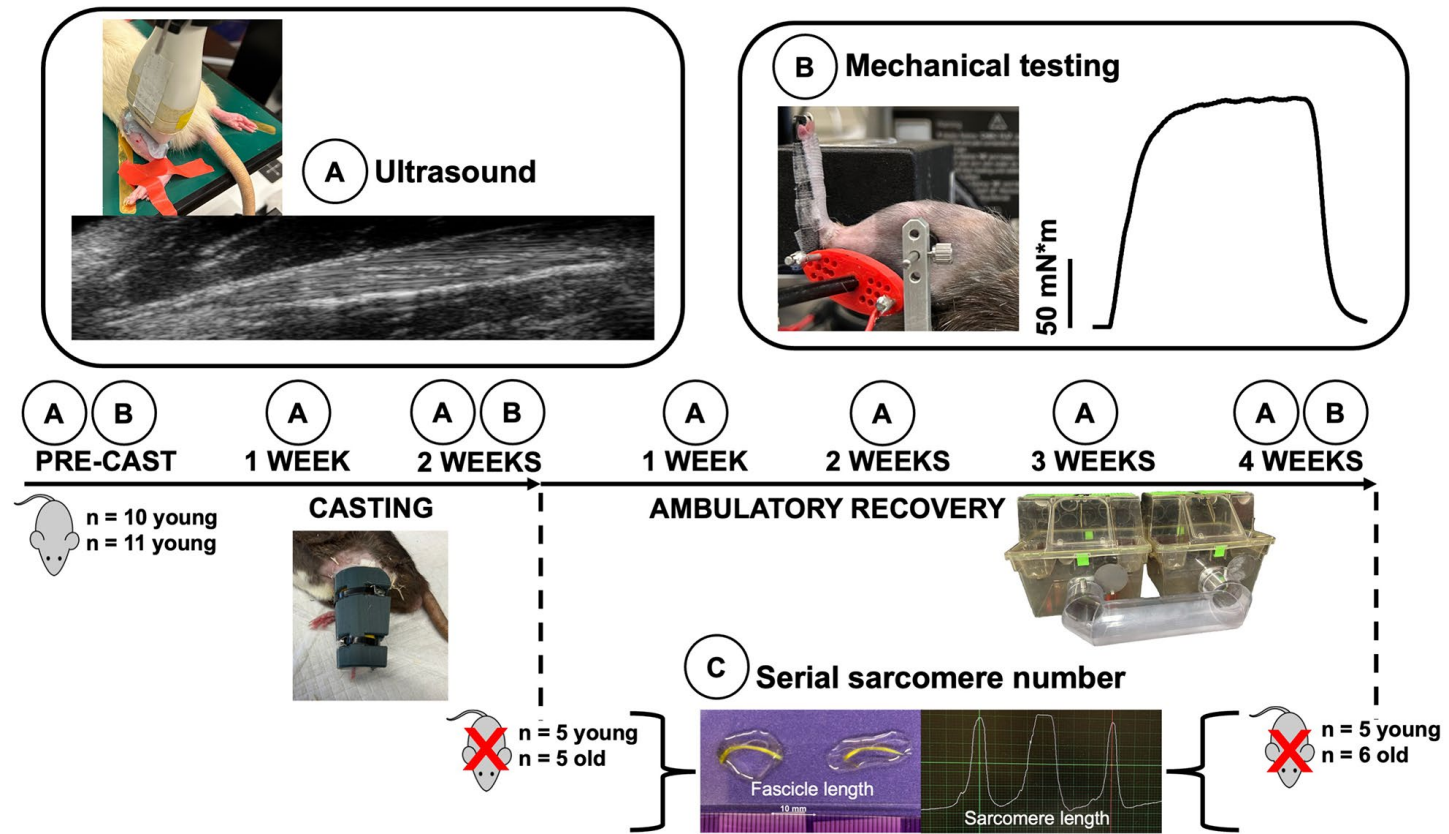
Experimental timeline. Young (*n* = 10) and old (*n* = 11) rats underwent 2 weeks of unilateral casting in full plantar flexion followed by 4 weeks of ambulatory recovery in a “double-cage” setup. Ultrasound measurements **(A)** were performed weekly. Mechanical testing **(B)** (construction of a passive and active torque-angle relationship) of the plantar flexors was performed pre-cast, after the 2-week casting intervention, and at 4 weeks of recovery. Post-cast, 5 young and 5 older adult rats were sacrificed for serial sarcomere number (SSN) measurements **(C)**. The remaining 5 young and 6 older adult rats were sacrificed after 4 weeks of recovery for SSN measurements

10 young adult (8 months) and 11 older adult (32 months) male Fisher 344/Brown Norway F1 rats were obtained (Charles River Laboratories, Senneville, QC, Canada). All protocols were approved by the University of Guelph’s Animal Care Committee (AUP #4905) and followed guidelines from the Canadian Council on Animal Care. Rats were housed at 23 °C in groups of two or three and given ad-libitum access to a Teklad global 18% protein rodent diet (Envigo, Huntington, Cambs., UK) and room-temperature water. 5 old and 5 young adult rats were sacrificed after the 2-week casting intervention, and the remaining 6 old and 5 young adult rats were sacrificed after the 4-week recovery period following cast removal. Ultrasound measurements on the casted soleus were obtained at 7 time points: no more than 1 week prior to the application of casts (pre-cast), 1 week into the casting intervention (1 wk cast), on the day of cast removal (post-cast), and 1, 2, 3, and 4 weeks following cast removal (1, 2, 3, and 4 wk recovery). Mechanical testing measurements were obtained at three time points: pre-cast, post-cast, and 4 wk recovery. While it is well-recognized that older adult rats partake in less voluntary physical activity than young [31], to encourage voluntary ambulation in both groups following cast removal, rats were housed in a double-cage setup including a large tube that connects two cages together (Fig. 1). Following sacrifice, the hindlimbs were fixed in formalin for subsequent determination of soleus SSN. In accordance with previous studies [19, 21, 35–37], the left leg served as the experimental leg while the right leg served as an internal control.

### Unilateral immobilization

Using gauze padding, vet wrap, and a 3D-printed brace and splint, the right hindlimb of each rat was casted in full plantarflexion for 2 weeks (Fig. 1). The toes were left exposed to monitor for swelling [37, 38]. Casts were inspected daily and repaired/replaced as needed. All casts were replaced 1 week into the casting period following the 1 wk cast ultrasound measurements. Since rats were free to walk around while wearing their casts, this intervention merely promoted disuse of the immobilized muscles rather than complete unloading such as that achieved with hindlimb suspension [39].

### Data acquisition during mechanical testing and training

A 701 C High-Powered, Bi-Phase Stimulator (Aurora Scientific, Aurora, ON, Canada) was used to evoke transcutaneous muscle stimulation via a custom-made electrode holder with steel galvanized pins situated transversely over the popliteal fossa and the calcaneal tendon (Fig. 1). During piloting, we determined this stimulation setup produced similar values of maximum isometric tetanic torque across repeated testing sessions, and produced plantar flexion torque values consistent with values in previous literature using direct nerve stimulation [40]. Torque, angle, and stimulus trigger data were sampled at 1000 Hz with a 605 A Dynamic Muscle Data Acquisition and Analysis System (Aurora Scientific, Aurora, ON, Canada) with a live torque tracing during all training and mechanical data collection sessions.

### Mechanical testing

The rats were anesthetized with isoflurane and positioned on a heated platform (37 °C) in a supine position. After shaving the leg completely of hair, the left leg was fixed to a force transducer/length controller foot pedal via tape, with the knee immobilized at 90°. Each mechanical testing session began with determination of the optimal current for stimulation (frequency = 100 Hz, pulse duration = 0.1 ms, train duration = 500 ms) at an ankle angle of 90° (ankle angle = angle between the tibia and the foot sole; full plantar flexion = 180°), which was the current used throughout the remainder of the session. This stimulation current was confirmed to maximally activate the plantar flexors with minimal spread to the (antagonist) dorsiflexors by completing another stimulation with the current further increased by 10 mA, in which a decrease in active torque was observed – indicating the current we used for the experiments involved minimal to no antagonist activation. 0.5-ms, 100-Hz isometric contractions were then completed at ankle angles of 70°, 80°, 90°, 100°, and 110°, each separated by 2 min of rest. Active torque was measured by subtracting the minimum value of torque at baseline (i.e., the passive torque) from the maximum value of total torque during stimulation [14, 41]. Following movement of the foot pedal, stimulation was preceded by 5 s of rest to reduce the impact of stress-relaxation on the measurements of passive torque.

### Ultrasonography

A UBM system (Vevo 2100; VisualSonics, Toronto, ON, Canada) operating at a centre frequency of 21 MHz was used to acquire images of the soleus, with a lateral resolution of 80 μm and an axial resolution of 40 μm [37, 42]. A 23-mm long probe was used, allowing acquisition of images displaying muscle fascicles from end to end. Image acquisition was optimized with an image depth of 14–15 mm for the soleus, allowing a maximum frame rate of 16 Hz [37]. Prior to image acquisition, rats were anesthetized using isoflurane. With the knee fully extended, tape was used to fix the left ankle at 90º with the rat in a prone position and the hindlimb externally rotated, with the probe overlying the lateral aspect of the posterior shank (Fig. 1). All ultrasound images were acquired by the same individual (A.H.). The probe position was carefully adjusted to obtain the clearest possible view of fascicles in all of the proximal, middle, and distal regions of the muscle. Throughout image acquisition, the probe was stabilized by a crane with fine-tune adjustment knobs, minimizing pressure and limiting the error associated with human movement.

Ultrasound images were analysed using ImageJ software [37, 43]. ImageJ’s multisegmented tool allowed careful tracing of the fascicle paths from end to end in measuring FL. Two measurements of FL and pennation angle were obtained from each of the proximal, middle, and distal regions of each muscle (i.e., *n* = 6 FL and pennation angle measurements per muscle) and averaged for the reporting of data, as we showed previously that soleus FL differs minimally across muscle regions [11, 37]. Pennation angle was defined as the angle between the fascicle and the aponeurosis at the fascicle’s distal insertion point. Three measurements of muscle thickness (proximal, middle, and distal) were also taken at the soleus mid-muscle belly (i.e., the thickest portion) and averaged [44, 45]. All FL, pennation angle, and muscle thickness measurements were obtained by the same experimenter (A.H.), who was always blinded to the results of a particular timepoint until all measurements were obtained. These measures were determined to have low coefficient of variation across three separate sessions in a previous study using the same ultrasound setup [37].

### Serial sarcomere number determination

Following their final mechanical testing session, rats were sacrificed via isoflurane anesthetization followed by CO_2_ asphyxiation. The hindlimbs were amputated, skinned with all muscles overlying the soleus removed, and fixed in 10% phosphate-buffered formalin with the ankle pinned at 90°. After fixation for 1–2 weeks, the soleus was dissected off the lower leg, weighed, then re-submerged in formalin until the commencement of SSN estimations. To commence the process of SSN estimations, the muscles were rinsed with phosphate-buffered saline, then digested in 30% nitric acid for 6–8 h to remove connective tissue and allow for individual muscle fascicles to be teased out [14, 46].

For each muscle, two fascicles were obtained from each of the proximal, middle, and distal regions (i.e., *n* = 6 fascicles total per muscle). SSN and FL values were averaged across these six fascicles for the reporting of data, as we showed previously that SSN differs minimally across region in the rat soleus [11, 37]. Dissected fascicles were placed on glass microslides (VWR International, USA), then FLs were measured using ImageJ software (version 1.53f, National Institutes of Health, USA) from pictures captured by a level, tripod-mounted digital camera, with measurements calibrated to a ruler in plane with the fascicles (Fig. 1). Sarcomere length (SL) measurements were taken at *n* = 6 different locations proximal to distal along each fascicle via laser diffraction (Coherent, Santa Clara, CA, USA) with a 5-mW diode laser (500 μm beam diameter, 635 nm wavelength) and custom LabVIEW program (Version 2011, National Instruments, Austin, TX, USA) [47] (Fig. 1), for a total of *n* = 36 SL measurements per muscle. For each fascicle, the six SL measurements were averaged to obtain a value of average SL. Given the laser diameter of ∼ 1 mm, one SL measurement itself represents an average of thousands of SLs. Our total quantity of SL and FL measurements is consistent with previous studies [14, 41, 46]. For each fascicle, the six SL measurements were averaged to obtain a value of average SL. Serial sarcomere number of each fascicle was calculated as:

$$\text{Serial sarcomere number} = \text{fascicle length} / \text{average sarcomere length}$$

Within each fascicle, SL standard deviation (SL SD) was also noted as an estimate of SL non-uniformity.

### Estimation of time-course SSN adaptations using ultrasound-derived FL

It is often difficult to measure the time course of SSN adaptations due to the invasive nature of SSN measurements, requiring separate groups of animals to be sacrificed at each time point. We recently verified a method of using ultrasonographic measurements of FL to estimate SSN adaptations in the rat soleus provided that a correction factor is applied [37]. Thus, as a final analysis to address our primary research question (age-related differences in SSN adaptations during and following immobilization), we used the ultrasound-derived measurements of FL to estimate the time course of SSN adaptations. To do this, we first calculated the ratio of the ultrasound-derived FL values to the measures of FL on dissected fascicles (this value was 1.04; see Results). This ratio was used as a correction factor to convert all ultrasound FL measurements to dissected FL measurements. Since SL did not differ between the post-cast and 4 wk recovery time points in old or young adult rats (see Results), SL values from the experimental leg were used for estimations of SSN post-cast and at 1, 2, and 3 wk recovery. To summarize, estimations of time-course changes in SSN using ultrasound-derived FL were done via the equation:

$$\text{Estimated SSN} = (\text{ultrasound-derived FL} / 1.04) / \text{estimated SL}$$

#### Determination of physiological cross-sectional area

To gain further insight on changes in muscle contractile tissue in parallel throughout the study, we calculated physiological cross-sectional area (PCSA, in cm^2^) using the Eq. [48]:

$$\:PCSA=\frac{Muscle\:mass\:\times\:\:\text{c}\text{o}\text{s}\left(pennation\:angle\right)}{Muscle\:density\:\times\:\:normalized\:FL}$$

This calculation was performed for control and casted muscles at post-cast and 4 wk recovery. Muscle wet weight was used as muscle mass. Pennation angle at pre-cast was used for pennation angle of control muscles. Muscle density was assumed to be 1.112 g/cm^3^ [48]. Normalized FL was calculated using FL of dissected fascicles and measured SL in the Eq. [48]:

$$\:Normalized\:FL=FL\:\frac{SLo}{measured\:FL}$$

SLo represents optimal SL of rat muscle at rest, assumed to be ∼ 2.7 μm based on previous literature [41, 49].

### Statistical analysis

All statistical analyses were performed in SPSS Statistics Premium 28. Normality of all data was confirmed using Shapiro-Wilk tests. To investigate baseline age-related differences in muscle structure, one-way analysis of variance (ANOVA) was used on pre-cast measurements for ultrasound data (ultrasound-derived FL, pennation angle, and muscle thickness) and on measurements from the control leg for dissected muscle data (muscle wet weight, PCSA, SSN, SL, SL SD, dissected FL). To investigate differences between time points in each age group for dissected muscle measurements, two-way repeated measures ANOVA (leg [control, casted] × timepoint [post-cast, 4 weeks recovery]) were employed. To investigate age-related differences in time-course changes of ultrasound measurements, two-way repeated measures ANOVA (age [young, old] × time [pre-cast, 1 week cast, post-cast, 1, 2, 3, 4 weeks recovery]) were employed. To investigate changes in the active and passive torque-angle relationships, three-way repeated measures ANOVA (age [young, old] × time [pre-cast, post-cast, 4 weeks recovery] × angle [70°, 80°, 90°, 100°, 110°]) were employed. Three-way ANOVA was used in these cases to allow us to investigate differences in the overall shapes (influenced by changes across different joint angles) of the active and passive torque-angle relationships. Lastly, to evaluate the estimated time-course changes in SSN (as estimated via ultrasound-derived FL) throughout the whole study, we applied a two way repeated measures ANOVA (age [young, old] × time point [pre-cast, post-cast, 1, 2, 3, 4 wk recovery]).

For all ANOVAs, a Greenhouse Geiser correction for Sphericity was applied. Where main effects or interactions were detected, two-tailed t-tests were used for pairwise comparisons, with a Sidak correction for multiplicity. Significance was set at α = 0.05. All values are reported as the mean ± standard deviation.

## Results

### Control/pre-cast age-related differences in soleus muscle morphology

Muscle wet weight, SSN, and dissected FL as measured at 90° were 8%, 11%, and 12% less, respectively, in the control legs of old compared to young adult rats (Table 1). SL and SL SD as measured at 90° did not differ between young and older adult rats (Table 1). From the ultrasound measurements at 90° pre-cast, FL and muscle thickness were 8% and 15% less, respectively in old compared to young adult rats, while pennation angle did not differ between old and young (Table 1). PCSA of the control legs did not differ between young and older adult rats (Table 1).

**Table 1:** Control/pre-cast soleus morphological properties in old compared to young adult rats

	Control leg or pre-cast	Young (*n* = 10)	Old (*n* = 11)	% Difference	One-Way ANOVA
Wet weight (mg)	Control leg	247.90 ± 18.10	227.67 ± 20.36	–8.16%*	**F** (1, 21) **= 5.739** ***P*** **= 0.027**
Serial Sarcomere Number	Control leg	5532 ± 115	4904 ± 242	–11.4%*	**F** (1, 21) **= 55.461** ***P*** **< 0.001**
Fascicle Length (dissected) (mm)	Control leg	12.1 ± 0.4	10.6 ± 0.4	–12.4%*	**F** (1, 21) **= 65.826** ***P*** **< 0.001**
Sarcomere Length (µm)	Control leg	2.18 ± 0.07	2.17 ± 0.07	N/A	F (1, 21) = 0.076 *P* = 0.786
Sarcomere Length Standard Deviation (µm)	Control leg	0.12 ± 0.05	0.13 ± 0.03	N/A	F (1, 21) = 0.980 *P* = 0.335
Fascicle Length (ultrasound) (mm)	Pre-cast	12.7 ± 0.4	11.7 ± 1.1	–7.9%*	**F** (1, 21) **= 7.089** ***P*** **= 0.015**
Pennation angle (°)	Pre-cast	7.2 ± 1.0	7.7 ± 0.7	N/A	F (1, 21) = 1.799 *P* = 0.196
Muscle thickness (mm)	Pre-cast	2.0 ± 0.1	1.7 ± 0.2	–15.0%*	**F** (1, 21) **= 9.349** ***P*** **= 0.006**
Physiological cross-sectional area (cm^2^)	Calculated via control leg and pre-cast values	0.148 ± 0.012	0.154 ± 0.014	N/A	F (1, 21) = 0.950 *P* = 0.342

*Difference between old and young (*P* **< 0.05); values are reported as mean ± standard deviation**

### Muscle wet weight and physiological cross-sectional area decreased with casting in young and older adult rats, then partially recovered in young adult rats but remained unrecovered in older adult rats

**Fig. 2:**
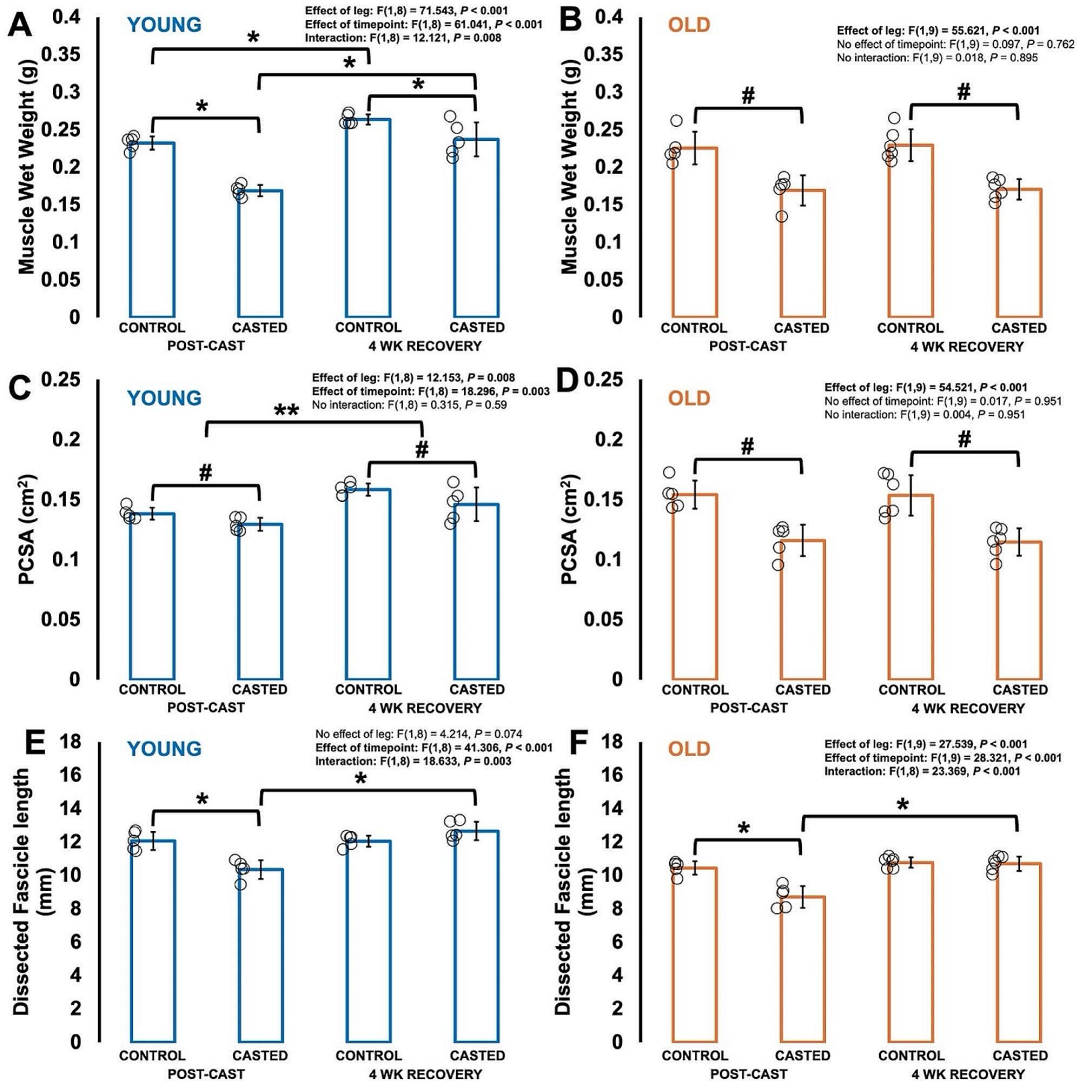
Differences in muscle wet weight (**A-B**), physiological cross-sectional area (PCSA) (**C-D**), and fascicle length of dissected fascicles as measured at 90° (**E-F**) at post-cast (*n* = 5 young, *n* = 5 old) and 4 weeks of recovery (*n* = 5 young, *n* = 6 old) in casted muscles compared to control muscles. Data are displayed as mean ± standard deviation. *Difference between indicated points (*P* < 0.05). **Effect of timepoint with control and casted legs combined (*P* < 0.05). #Effect of leg with timepoints combined (*P* < 0.05)

For muscle wet weight of young adult rats, there was a leg × timepoint interaction (Fig. 2A). Post-cast, young adult rats had a 27% lower muscle wet weight in the casted compared to control leg (*P* < 0.001). At 4 wk recovery, muscle wet weight of young adult rats was then greater in the casted leg compared to post-cast (*P* < 0.001), however, was still less than that of the control leg by 10% (*P* = 0.008) (Fig. 2A). By contrast, older adult rats only showed an effect of leg for muscle wet weight such that muscle wet weight of the casted leg was 25% less than that of the control leg both post-cast and at 4 wk recovery (Fig. 2B).

For PCSA of young adult rats, there was an effect of leg such that PCSA of the casted leg was 7% less than the control leg at both post-cast and recovery (Fig. 2C). There seemed to be some recovery of PCSA in young adult rats, however, there was also an effect of timepoint with PCSA at 4 wk recovery being 12% greater than at post-cast with both legs combined (Fig. 2C). For PCSA of older adult rats, there was only an effect of leg such that PCSA was 25% less in the casted leg compared to the control leg at both post-cast and 4 wk recovery (Fig. 2D).

### Dissected fascicle length decreased with casting and recovered following cast removal in both young and older adult rats

For dissected FL as measured at 90°, there were leg × time interactions for both young and old, which showed similar decreases in FL from the control to the casted leg post-cast (young: -14%, *P* = 0.002; old: -17%, *P* < 0.001) (Fig. 2E-F). FL of the casted leg then increased from post-cast to 4 wk recovery in young (*P* < 0.001) and old (*P* < 0.001) and did not differ from the control leg (young: *P* = 0.148; old: *P* = 0.766) (Fig. 2C).

### Sarcomere length and sarcomere length standard deviation were greater in the casted leg than the control leg in young and older adult rats at both post-cast and 4 wk recovery

In young and older adult rats, SL as measured at 90° showed no effects of time but did show effects of leg, with SL being longer in the casted leg compared to the control leg in young (control: 2.18 ± 0.07 μm; casted: 2.38 ± 0.12 μm) and older adult rats (control: 2.17 ± 0.07 μm; casted: 2.25 ± 0.08 μm) regardless of time point (Supplemental Figure S1A-B). The same pattern was observed for SL SD, with no effects of time but effects of leg that showed greater SL SD (young: +59%; old: +32%) in the casted than the control leg regardless of time point (Supplemental Figure S1C-D).

### Ultrasound-derived fascicle length, muscle thickness, and pennation angle decreased with casting in young and older adult rats, then fascicle length and muscle thickness recovered sooner in young than older adult rats, and pennation angle did not recover

**Fig. 3:**
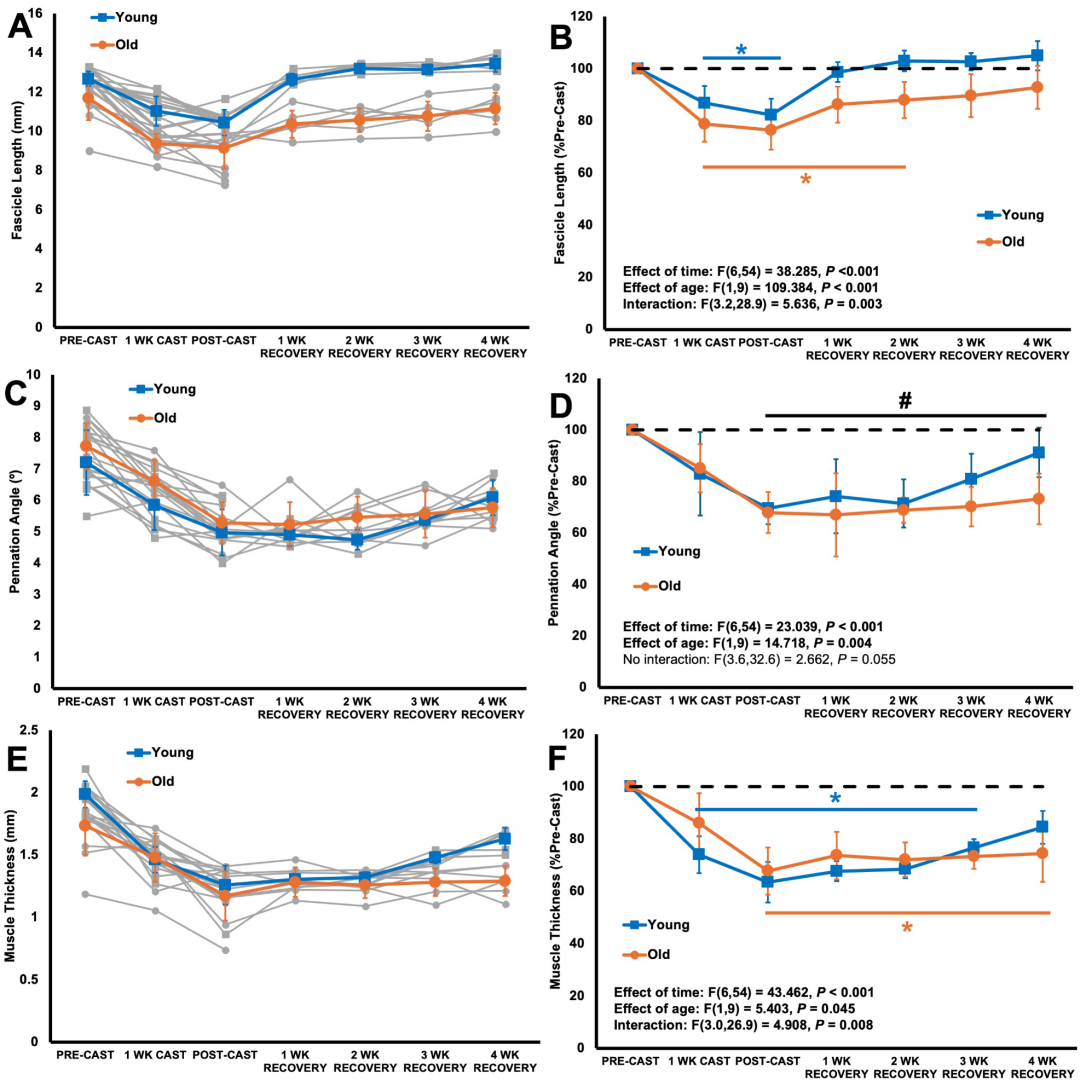
Changes in ultrasound-derived fascicle length (**A-B**), pennation angle (**C-D**), and muscle thickness (**E-F**) throughout casting (*n* = 10 young, *n* = 11 old) and recovery (*n* = 5 young, *n* = 6 old), with half the rats sacrificed post-cast and the remaining half sacrificed after 4 weeks of recovery. Left graphs display absolute values with individual data (grey lines), on which statistical analyses were performed. Right graphs show data normalized to pre-cast. Data are displayed as mean ± standard deviation. *Difference from pre-cast (*P* < 0.05). #Difference from pre-cast with old and young combined (*P* < 0.05), as there was no interaction

For ultrasound-derived FL, there was an age × time interaction (Fig. 3A-B). Both old and young adult rats showed reductions in FL at 1 wk cast (both *P* < 0.001) and post-cast (*P* = 0.002–0.005), with young decreasing by 15% and old by 22% (Fig. 3B). Ultrasound-derived FL recovered sooner in young adult rats, as they no longer differed from pre-cast at 1 wk recovery (*P* = 1.000), while older adult rats differed from pre-cast at 1 wk (*P* = 0.009) and 2 wk (*P* = 0.021), then recovered at 3 wk (*P* = 0.096 compared to pre-cast) (Fig. 3B).

For pennation angle, there was an effect of time such that both young and older adult rats had a 35% reduction in pennation angle at post-cast (*P* < 0.001) (Fig. 3C-D), and did not recover by 4 wk (*P* < 0.001–0.007 throughout recovery period).

For muscle thickness, there was an age × time interaction (Fig. 3E-F). At 1 wk cast, muscle thickness decreased in young adult rats (-23%, *P* = 0.020) but not older adult rats (*P* = 0.668). At post-cast, young decreased further (-37%, *P* < 0.001) and old decreased compared to pre-cast (-30%, *P* = 0.002) (Fig. 3F). Muscle thickness of young adult rats differed from pre-cast at 1, 2, and 3 wk recovery (all *P* < 0.001) then no longer differed from pre-cast at 4 wk (*P* = 0.173), while muscle thickness of older adult rats remained unrecovered throughout the recovery period (*P* < 0.001–0.008 compared to pre-cast) (Fig. 3F).

### Serial sarcomere number decreased similarly during casting in young and older adult rats, then recovered faster in young compared to older adult rats

**Fig. 4:**
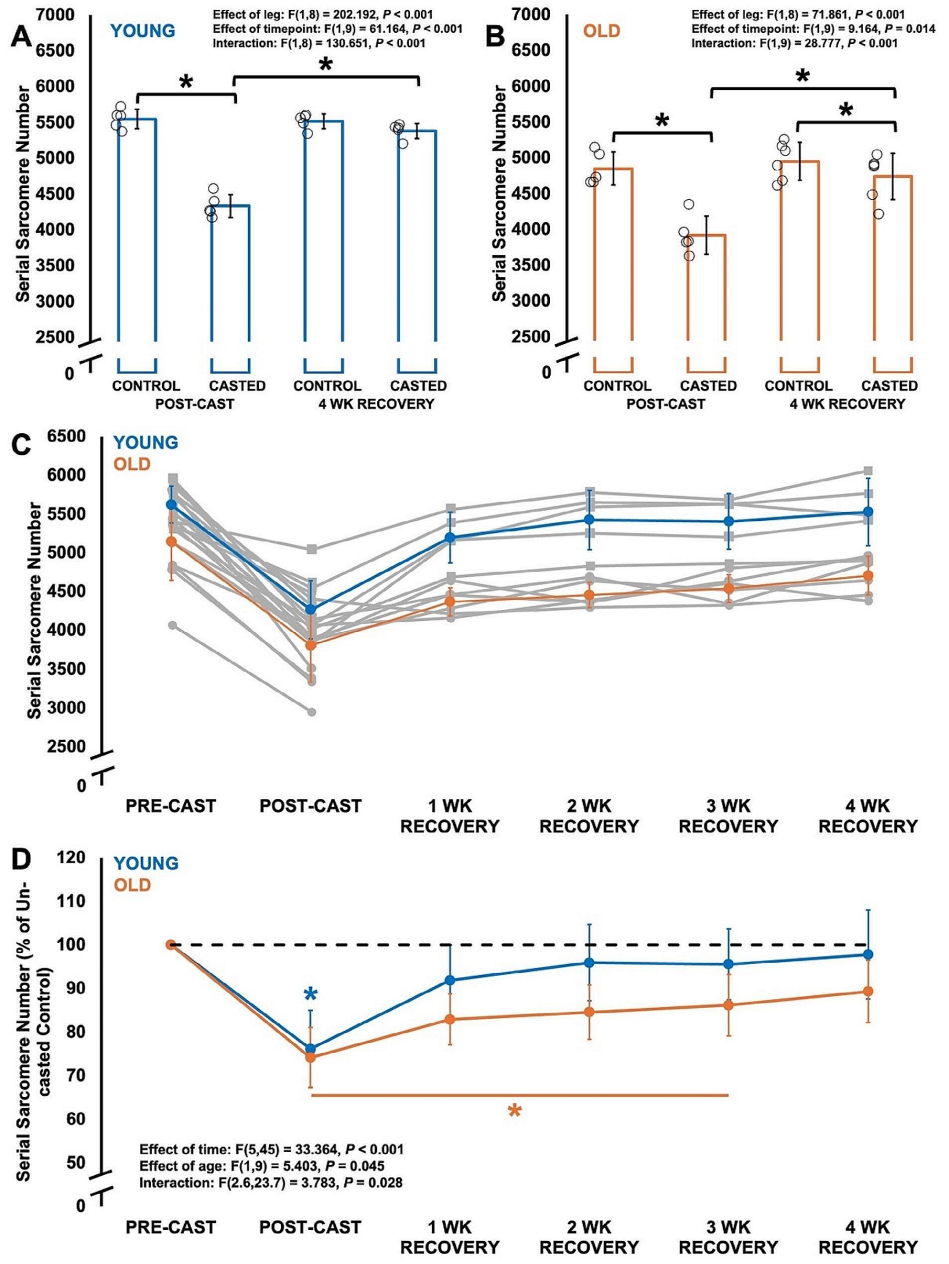
**A-B.** Differences in serial sarcomere number at post-cast (*n* = 5 young, *n* = 5 old) and 4 weeks of recovery (*n* = 5 young, *n* = 6 old) compared to control muscles. Data are displayed as mean ± standard deviation. *Significant difference between indicated points (*P* < 0.05). **C-D.** Time-course changes in serial sarcomere number throughout casting (*n* = 10 young, *n* = 11 old) and recovery (*n* = 5 young, *n* = 6 old), estimated using ultrasound-derived fascicle length. C shows absolute values with individual data (grey lines), on which statistical analyses were performed. D shows data normalized to pre-cast. Data are displayed as mean ± standard deviation. *Difference from pre-cast (*P* < 0.05)

For SSN, there was a leg × time interaction for both young and old (Fig. 4A-B). Young (-22%, *P* < 0.001) and old (-19%, *P* < 0.001) showed similar reductions in SSN from the control to casted leg at post-cast (Fig. 4A). SSN of the casted leg in both young (*P* < 0.001) and old (*P* < 0.001) then increased from post-cast to 4 wk recovery, however, in young the casted and control legs no longer differed in SSN (*P* = 0.084) (Fig. 4A), while SSN of the casted leg in older adult rats was still 4% less than the control leg (*P* = 0.046) (Fig. 4B).

Ultrasound-derived FL was on average 1.04 times FL of dissected fascicles from the same muscles (Supplemental Figure S2). When using ultrasound-derived FL to better estimate time-course changes in SSN, there was an age × time interaction (Fig. 4C-D). In both young and older adult rats, SSN decreased 24% from pre- to post-cast (*P* < 0.001). Young adult rats’ SSN no longer differed from pre-cast at 1 wk recovery and onwards (*P* = 0.381-1.000) (Fig. 4D). By contrast, older adult rats’ SSN remained lower than pre-cast at 1 to 3 wk recovery (*P* = 0.006–0.036), then recovered at 4 wk (*P* = 0.240 compared to pre-cast) (Fig. 4C). These data suggest that old and young both recovered SSN, however, young adult rats recovered faster (1 week following cast removal) than older adult rats (4 weeks following cast removal).

### Active torque decreased and passive torque increased following casting in young and older adult rats, then active torque recovered following cast removal in only young adult rats, while passive torque remained elevated following casting in both young and older adult rats

**Fig. 5:**
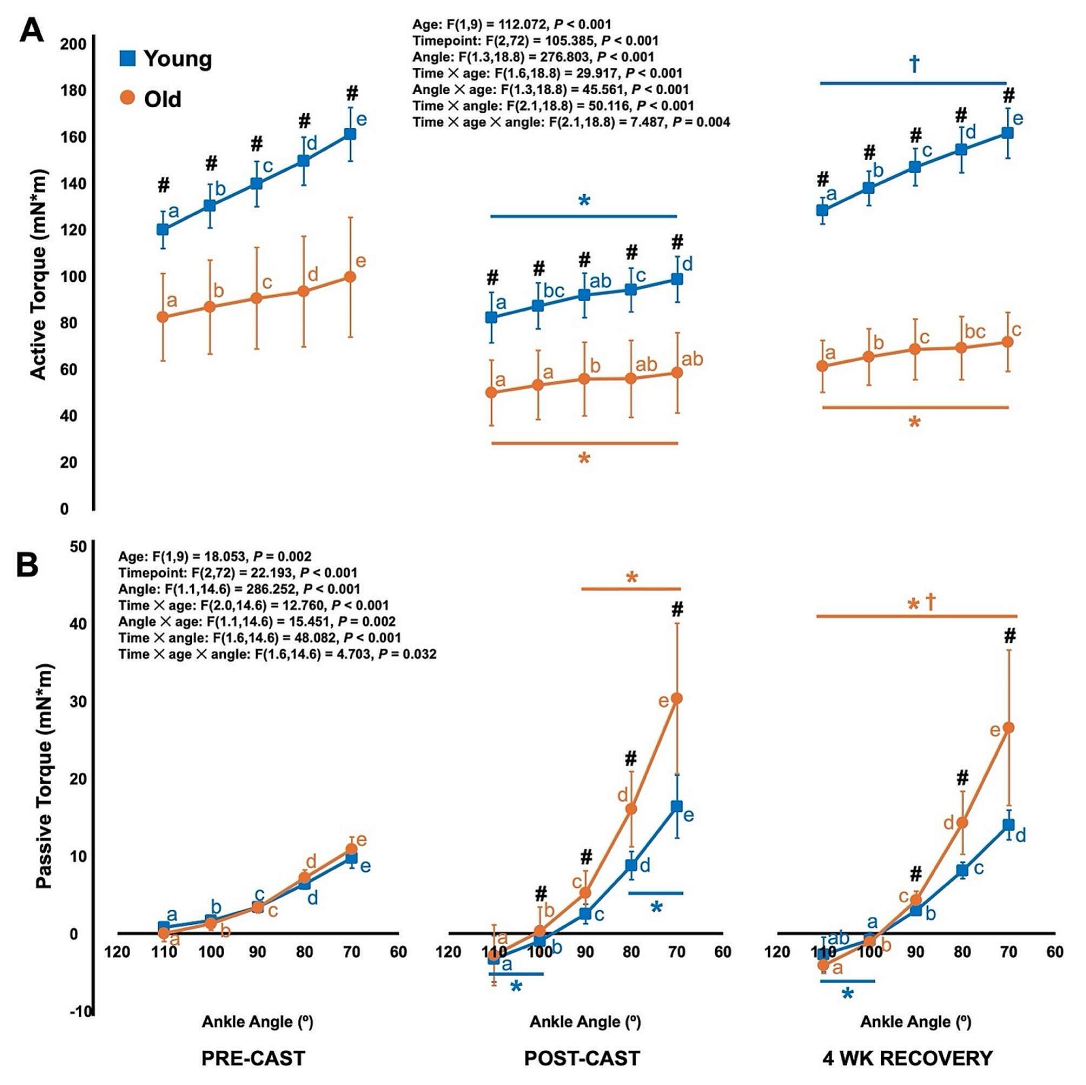
Differences in the active (**A**) and passive (**B**) torque-angle relationships between young (*n* = 10 pre- and post-cast; *n* = 5 at 4 wk recovery) and older adult rats (*n* = 11 pre- and post-cast; *n* = 6 at 4 wk recovery) and across time. *Difference from pre-cast (*P* < 0.05). †Difference from post-cast (*P* < 0.05). #Difference between young and old (*P* < 0.05). Same letters denote no significant difference within a time point and age group (*P* > 0.05)

For the active torque-angle relationship, there was an age × time × angle interaction (Fig. 5A). Older adult rats produced lower active torque than young at all angles pre-cast (-31 to -38%, all *P* = < 0.001), post-cast (-39 to -41%, all *P* < 0.001), and at 4 wk recovery (-52 to -56%, all *P* < 0.001) (Fig. 5A). In young adult rats, active torque decreased at all angles from pre to post-cast (-31 to -39%, all *P* < 0.001), notably to values that were almost identical to those of older adult rats pre-cast (Fig. 5A). Active torque then increased at all angles from post-cast to 4 wk recovery (+ 56–64%, all *P* < 0.001), no longer differing from pre-cast (*P* = 0.090–0.980). At pre-cast and 4 wk recovery, active torque differed among all angles (all *P* < 0.001), while at post-cast active torque was statistically the same between some angles (*P* = 0.052–0.969) (Fig. 5A), suggesting a shift of the torque-angle relationship’s plateau region to a more plantar flexed angle from pre to post-cast, then a return to a more dorsiflexed angle at 4 wk recovery.

In older adult rats, active torque also decreased at all angles from pre to post-cast (-39 to -41%, all *P* < 0.001) (Fig. 5A). At 4 wk recovery, active torque at all angles was still less than pre-cast (-24 to -28%, all *P* < 0.001), and did not differ from post-cast (*P* = 0.114–0.590), indicating minimal recovery of active torque (Fig. 5A). Furthermore, like in young adult rats, active torque differed among all angles pre-cast (*P* < 0.001–0.005), suggesting optimal torque occurred at a more dorsiflexed angle, but post-cast active torque was the same among most angles (*P* = 0.052–0.995), suggesting a shift of the plateau region to a more plantar flexed angle (Fig. 5A). At 4 wk recovery, there seemed to be some reversal of this plateau region shift, with torque at 70° being greater than torque at 110° (*P* = 0.008) and 100° (*P* = 0.017) (Fig. 5A).

For the passive torque-angle relationship, there was an age × time × angle interaction (Fig. 5B). In both young and older adult rats at all time points, passive torque consistently increased as the ankle angle became more dorsiflexed (*P* < 0.001–0.024) (Fig. 5B). Pre-cast, old and young adult rats did not differ in passive torque at any angles (*P* = 0.086–0.623). From pre- to post-cast, passive torque of young adult rats increased at 70° (+ 68%, *P* = 0.006) and 80° (+ 37%, *P* = 0.022), and of older adult rats increased at 70° (+ 180%), 80° (+ 124%), and 90° (+ 55%) (all *P* < 0.001) (Fig. 5B). This casting-related increase in passive torque was more pronounced in older adult rats, as post-cast, older adult rats produced greater passive torque than young at angles 70° to 90° (+ 83–109%, *P* < 0.001–0.015) (Fig. 5B). At 4 wk recovery, passive torque of young adult rats no longer differed from pre-cast at 70° (*P* = 0.482) and 80° (*P* = 0.452), however, also did not differ from post-cast (*P* = 0.165–0.448), suggesting incomplete recovery (Fig. 5B). Passive torque of older adult rats at 4 wk recovery decreased compared to post-cast at 70° (*P* = 0.016), 80° (*P* = 0.023), and 90° (*P* = 0.019), but these were still elevated compared to pre-cast (+ 27–144%, *P* < 0.001–0.007) (Fig. 5B). Furthermore, older adult rats still produced greater passive torque than young at angles 70° to 90° at 4 wk recovery (+ 43–90%, *P* = 0.010–0.049).

It is also important to note that passive torque in young adult rats became more negative at 100° and 110° post-cast and at 4 wk recovery (*P* < 0.001–0.012), and in older adult rats at 4 wk recovery (*P* < 0.001–0.001) (Fig. 5B), suggesting a steepening of the dorsiflexor passive torque-angle curve as well.

## Discussion

The purpose of this study was to investigate age-related differences in muscular adaptations during casting in a shortened position and subsequent recovery, with a particular focus on the regulation of longitudinal muscle growth governed by SSN. We found similar magnitudes of decrease between young and older adult rats for SSN, muscle wet weight, muscle thickness, pennation angle, and isometric active torque production after 2 weeks of casting, but there was a greater increase in passive torque in older adult rats compared to young adult rats. Following cast removal, young adult rats exhibited quicker recovery of SSN, PCSA, muscle thickness, muscle wet weight, and maximum isometric torque than old. Additionally, voluntary ambulation provided a stronger stimulus for SSN growth than growth of parallel muscle morphology (PCSA and muscle thickness) in both young and older adult rats, highlighting the urgency that the system places on regulating muscle length through the addition of SSN.

### Age-related differences in baseline muscle morphology

The control soleus of older adult rats had a lower muscle wet weight (–8%) and SSN (–11%) compared to young. Since SL as measured at an ankle angle of 90° did not differ between young and old, the lower SSN in older adult rats reflected the shorter FL of dissected fascicles (–11%) as measured at 90°. Ultrasonographic measurements at the pre-cast timepoint showed similar age-related differences, with muscle thickness (–15%) and FL (–8%) both being smaller in older adult rats. These age-related changes in muscle morphology are consistent with previous reports in F344/BN rats [10, 40, 50, 51], and collectively reflect an age-related loss of muscle contractile tissue, which is driven largely by motor neuron loss [52] and increased rates of protein degradation and muscle fibre cell apoptosis [50, 53]. However, PCSA of the control legs did not differ between young and older adult rats. PCSA represents contractile tissue in parallel at the whole-muscle level, and is thus considered proportional to maximum force production [54]. Since older adult rats were 30–40% weaker than young adult rats, but PCSA did not differ, that indicates a reduced muscle quality in the older adult rats. Reduced muscle quality in old age has been observed previously [55, 56], and underscores changes in the muscle’s intrinsic contractile machinery such as reduced myosin protein content or oxidation of myosin, which reduces crossbridge formation/force [57, 58].

Notably, ultrasound-derived FL measurements underestimated the difference in soleus SSN between young and older adult rats by ∼ 3%. This finding aligns with our previous study that showed ultrasound-derived FL alone (i.e., without a measurement of SL), while often used as an in-vivo proxy of SSN, does not perfectly reflect actual SSN adaptations due to limitations associated with assuming SL, intramuscular connective tissue, and the two-dimensional nature of ultrasound scans [37]. This disconnect between ultrasound-derived FL and actual SSN likely explains why some studies in humans have observed age-related differences in ultrasound-derived FL [6, 16, 59–68] while others observed no differences [69–75]. Our results are consistent with previous studies that observed 7–37% lesser SSN in old than young adult rats and mice [9, 11, 76].

### Age-related differences in muscular adaptations to casting

Several studies on humans and rodents have shown that older individuals experience similar or smaller magnitude losses of muscle mass and strength compared to young following immobilization [32–34, 50, 77–80]. Aligning with those studies, we observed similar magnitude reductions in muscle wet weight (–25%), muscle thickness (–30% to − 37%), and maximum plantar flexor torque (–40%) between young and older adult rats following 2 weeks of immobilization in a shortened position. We now show that SSN loss (–24%) is also similar between young and older adult rats following 2 weeks of immobilization in a shortened position. Across studies on the regulation of SSN, it is generally accepted that subtraction of serial sarcomeres during casting in a shortened position occurs to reduce sarcomeric compression and restore the original resting SL for optimal force production in that shortened position [13, 18–21]. Interestingly, young adult rats exhibited less of a reduction in PCSA (–7%) compared to older adult rats (–25%) following immobilization. Therefore, it seems the loss of contractile tissue in parallel during immobilization was exacerbated by old age while the loss of contractile tissue in series was not.

The loss of contractile tissue in parallel indicated by the reductions in PCSA, muscle thickness, and pennation angle corresponded to the reductions in maximum isometric strength in both young and older adult rats. Additionally, the change in shape of the active torque-angle relationship from pre- to post-cast reflected the loss of SSN. Pre-cast, the ankle angles we assessed (70° to 110°) represented the ascending limb of the torque-angle relationship as evidenced by the significant differences in torque between each angle (Fig. 5A first panel). Post-cast, however, across the same range of joint angles, we were testing more on the torque-angle relationship’s plateau region as there was more homogeneity in torque between angles (i.e., a flatter torque-angle relationship; Fig. 5A second panel). This change represents a shift in optimal angle to a more shortened position, which is consistently observed alongside a decrease in SSN to optimize myofilament overlap and force production at the new resting muscle length [19–21].

While passive tension generated within the sarcomere (i.e., by the protein titin) contributes to some of the passive torque exhibited at the joint level, passive tension generated by the extracellular matrix (ECM) seems to contribute more [81, 82]. Regardless, from pre- to post-cast, both young and old exhibited increases in passive torque in accordance with previous studies [18, 19], however, the increase was more pronounced in old, with older adult rats producing 109% greater passive torque than young at 70° (i.e., the most stretched angle tested). This age-related greater increase in passive torque with casting largely reflects an age-related greater collagen accumulation and in particular greater collagen crosslinking in the ECM [83–87]. The loss of SSN may have also contributed to the greater passive torque, as SL at 90° was 4–9% longer in the casted than the control leg at both post-cast and 4 wk recovery (Supplemental Figure S1). Longer resting SLs in the casted soleus would increase passive tension, which could elevate total plantar flexor passive torque. However, based on our passive torque data following the recovery period (discussed below) and the age-related ECM changes observed in previous studies, it is likely that ECM adaptations contributed more than SSN adaptations to these immobilization-induced changes in the passive torque-angle relationship.

### Age-related differences in muscular adaptations during recovery from casting

Older adult rats exhibited slower or incomplete recovery compared to young rats for almost every variable measured. By 4 wk recovery, muscle wet weight of young adult rats recovered to 90% of control values, but in older adult rats remained unrecovered at 75%, about the same as that observed post-cast (Fig. 2A-B). Similarly, PCSA of young adult rats increased 12% from post-cast to 4 wk recovery, but in older adult rats was 25% reduced compared to the control leg at both post-cast and 4 wk recovery (Fig. 2C-D). Muscle thickness also recovered by 4 wk in young adult rats but remained unrecovered in older adult rats. As PCSA and muscle thickness are associated with maximal force production [54], it is understandable that maximal isometric torque of young adult rats fully recovered by 4 wk but for older adult rats remained 24–28% depressed compared to pre-cast and did not differ from post-cast. An age-related slower recovery of muscle mass and maximal isometric strength was also observed previously in studies on rodents and humans [33, 34, 51].

Previous studies on young rodents have shown that SSN can recover within 3 weeks following cast removal [24–26]. By using ultrasound-derived FL to estimate time-course changes in SSN, we demonstrated that SSN likely recovers even sooner, at least 1 week following cast removal. The adaptability of SSN during recovery following disuse, like the other measures described above, was age dependent. SSN of older adult rats appeared to recover at 4 wk rather than 1 wk recovery (Fig. 4D). With that said, this may not have represented full recovery for older adult rats, as SSN of the casted leg was still significantly 4% less than the control leg at 4 wk recovery in older adult rats (Fig. 4B). While a 4% difference in SSN may seem trivial, such a small difference could still be meaningful for older adults who have already lost contractile tissue due to aging. Regardless, the recovery of contractile tissue in series (i.e., SSN) occurred faster than our measure of contractile tissue in parallel (i.e., PCSA) following cast removal in both young and older adult rats. From these findings, it appears that early on during remobilization, the stimulus for SSN growth (stretching of muscle fascicles while walking) was stronger than the stimulus for parallel growth (loading of the muscle) [54]. Somewhat similar findings have been observed previously. During 2 weeks of immobilization followed by 4 weeks of retraining in young (24 years) and older adult (67 years) men, Hvid et al. [79] observed full recovery of fibre CSA in young men, but no recovery of type I and IIa fibre CSA in older men. It is possible that during muscle growth, the system places a greater emphasis on building sarcomeres aligned in series before in parallel. Spletter et al. [88] observed this stepwise process of muscle growth in developing *Drosophila* flight muscle, with SSN growing up until 60 h following puparium formation and plateauing thereafter, then growth of fibre CSA not occurring until 60 h following puparium formation and onwards.

Changes in the shape of the active torque-angle relationship at 4 wk recovery also reflected the observed SSN adaptations. The active torque-angle relationship of young returned to its original shape of the ascending region (Fig. 5A third panel), which aligns with the restoration of SSN. Old’s active torque-angle relationship retained a flatter appearance, but with less homogeneity in active torque between angles than at post-cast (e.g., with torque at 70° differing from 100° and 110°), suggesting a partial shift back toward the original optimal angle. The passive torque-angle relationships at 4 wk recovery, by contrast, were similar to post-cast for both old and young adult rats. Since SSN recovered in both groups, these steeper passive torque-angle relationships at 4 wk of recovery are likely associated with a lack of reversal of ECM adaptations.

Older adult rats partake in less voluntary physical activity than young adult rats [31], and reduced activity would limit mechanical loading on the hindlimb and the associated stimuli for muscle growth. Therefore, the present study’s design did not separate the contributions of an age-related reduction in voluntary activity and aging itself on the older adult rats’ slower recovery following disuse. With that said, several factors in aged muscle could limit the stimuli and signalling for SSN regrowth following disuse. Most notably, Horner et al. [27] recently showed that greater ECM-associated passive forces in muscles of older adult rats limit muscle excursions during walking and the forces generated at the ends of the range of motion. Such a reduced excursion could have resulted in a weaker stimulus for serial sarcomere addition [89] in our older adult rats during the recovery period. Additionally, Fuqua et al. [51] found that translational capacity and myofibrillar protein synthesis were not impaired in older adult rats during recovery from hindlimb unloading, thus they attributed the inability to recover muscle mass to higher rates of protein degradation. Regarding specifically SSN, downregulation of MuRF1 and the ubiquitin-proteasome system (a regulator of protein degradation) contributes to serial sarcomerogenesis [90], and greater MuRF activity has been reported in older individuals [91], especially in more severe cases of sarcopenia [92]. Collectively, greater protein degradation in old age could act against the synthesis of contractile proteins, slowing recovery of SSN. Satellite cells of aged muscle also exhibit dysfunction [93–95] and blunted responsiveness to mechanical stimuli [8, 96] that limit the capacity for muscle regeneration, including during recovery from disuse [33]. Lastly, older adult rats are more susceptible to muscle damage during recovery from disuse as compared to young adult rats [97], and this greater incurrence of muscle damage may result in a longer recovery period to fully restore contractile tissue [11, 98].

### Methodological considerations and future directions

We did not perform ultrasound measurements on the un-casted soleus, therefore, any potential compensatory adaptations in the un-casted leg were not accounted for. With that said, we previously observed no changes in soleus FL of the un-casted leg during 2 weeks of casting, as well as no differences in FL between the right and left soleus at pre-cast [37], so compensatory architectural adaptations in the un-casted soleus were likely minimal or absent in the present study. In contrast to the loss of SSN when immobilizing in a shortened position as shown in the present study, immobilizing muscle in a stretched position often induces an increase in SSN in young healthy rodents [13, 19, 38, 90]. As a future direction of this work, it would be interesting to also investigate age-related differences in SSN adaptations during immobilization in a stretched position. Additionally, the present study chose to assess the soleus because it is a uni-articular muscle, crossing only the ankle, and it was easier to keep the ankle consistently fully plantar flexed with our cast than it was to control knee angle (which other plantar flexors, the gastrocnemii and plantaris, also cross). The soleus is a primarily slow-type muscle [99], but fibre-type dependent differences in recovery of fibre CSA following disuse in aged muscle have been noted previously [79]. It would therefore be interesting to also investigate age-related differences in SSN adaptations during immobilization and recovery in mixed-fibred (medial gastrocnemius) or fast-fibred (plantaris) plantar flexor muscles [99]. Lastly, the nature of measuring SSN (requiring longitudinally dissected fascicles measured end to end) prevented us from obtaining muscle cross-sections for measurements of fibre CSA (i.e., sarcomeres in parallel). While we used PCSA (alongside measures of muscle thickness and maximum isometric torque) as an indicator of changes in whole-muscle contractile tissue in parallel, analysis of fibre CSA is warranted in future studies to gain a deeper understanding of age-related differences in the regulation of sarcomeres in series and in parallel.

## Conclusion

Here we showed for the first time that longitudinal muscle morphology, specifically the regulation of serially aligned sarcomeres, adapts more rapidly than parallel muscle morphology during recovery from immobilization in young and older adult rats. While SSN recovered slower in older adult rats compared to young, this recovery was still quicker than that observed for PCSA and muscle thickness, hence older adult rats retain a better ability to recover contractile tissue in series than in parallel. While this faster recovery of SSN did not rescue maximal force production, it did partially rescue the shape of the active torque-angle relationship. This rapid recovery of SSN represents critical early adaptive mechanisms driving longitudinal muscle growth to maintain functional capacity in older adults. Collectively, the faster recovery of SSN compared to parallel muscle morphology raises two important recommendations: (1) SSN is highly adaptable in aged muscle, therefore, training interventions targeting primarily SSN (e.g., eccentric resistance training) may have elevated success for improving mechanical performance following immobilization in older adults early in the rehabilitation process; and (2) since parallel muscle morphology recovers slower following disuse, parallel muscle morphology should be emphasized long-term during rehabilitation to fully restore functional capacity. Decreases in FL as measured by ultrasound (which could be driven by reduced SSN as shown here) have been observed in humans following bed rest, which places lower limb muscles in slightly shortened positions [100, 101]. Furthermore, muscle disuse in hemiparetic stroke patients resulted in 21% loss of biceps brachii SSN [102]. It is therefore likely that SSN loss occurs during real-world situations of disuse in elderly humans, and our findings highlight the highly adaptive potential of SSN early on in recovery from such disuse.

### Electronic supplementary material

Below is the link to the electronic supplementary material.

Supplementary Material 1

Supplementary Material 2: **figure S1** Differences in sarcomere length (A-B) and sarcomere length standard deviation (SD) (estimate of sarcomere length non-uniformity) (C-D) between control and casted legs in young (*n* = 10) and old (*n* = 11) rats, with post-cast and 4 wk recovery time points combined because there were effects of leg but not time. Data are displayed as mean ± standard deviation. *Difference between indicated points (*P* < 0.05). **Figure S2**: Ratio of fascicle length (FL) measured using ultrasound to FL measured on dissected fascicles from the same muscles.

## Data Availability

All data generated or analyzed during the study are available from the corresponding author upon request.
