# Supplementary figures and images for: Age-related differences in the loss and recovery of serial sarcomere number following disuse atrophy in rats
Source: Skelet Muscle. 2024 Aug 2;14:18. doi: 10.1186/s13395-024-00351-5 (PMC11295870; doi:10.1186/s13395-024-00351-5)

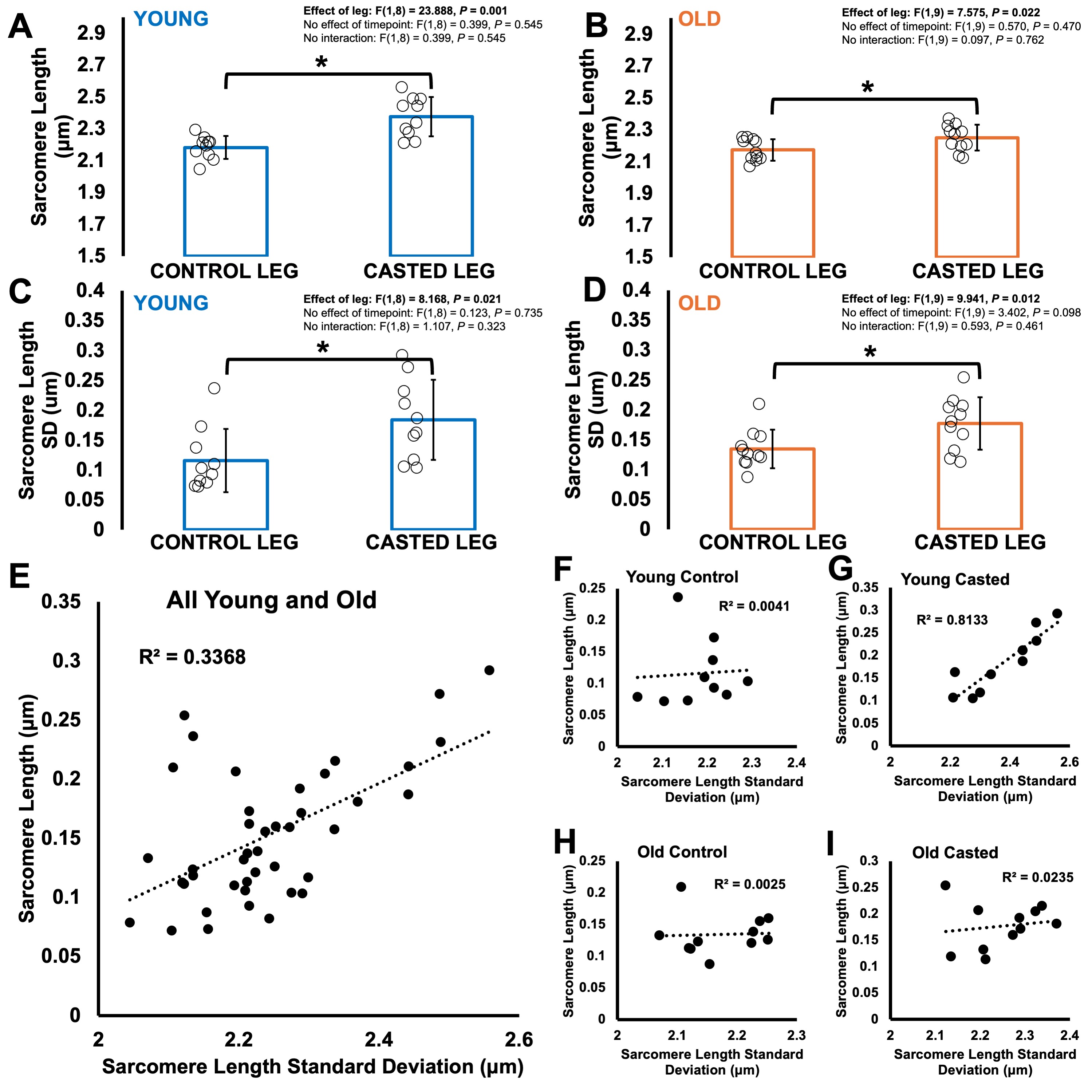

Supplement: Supplementary file 1 — Supplementary Material 1 [file 13395_2024_351_MOESM1_ESM.jpg]
