# Supplementary material for: Age-related differences in the loss and recovery of serial sarcomere number following disuse atrophy in rats
Source: Skelet Muscle. 2024 Aug 2;14:18. doi: 10.1186/s13395-024-00351-5 (PMC11295870; doi:10.1186/s13395-024-00351-5)
Supplement: Supplementary file 2 — Supplementary Material 2: figure S1 Differences in sarcomere length (A-B) and sarcomere length standard deviation (SD) (estimate of sarcomere length non-uniformity) (C-D) between control and casted legs in young (n = 10) and old (n = 11) rats, with post-cast and 4 wk recovery time points combined because there were effects of leg but not time. Data are displayed as mean ± standard deviation. *Difference between indicated points (P < 0.05). Figure S2: Ratio of fascicle length (FL) measured using ultrasound to FL measured on dissected fascicles from the same muscles. [file 13395_2024_351_MOESM2_ESM.docx]

**
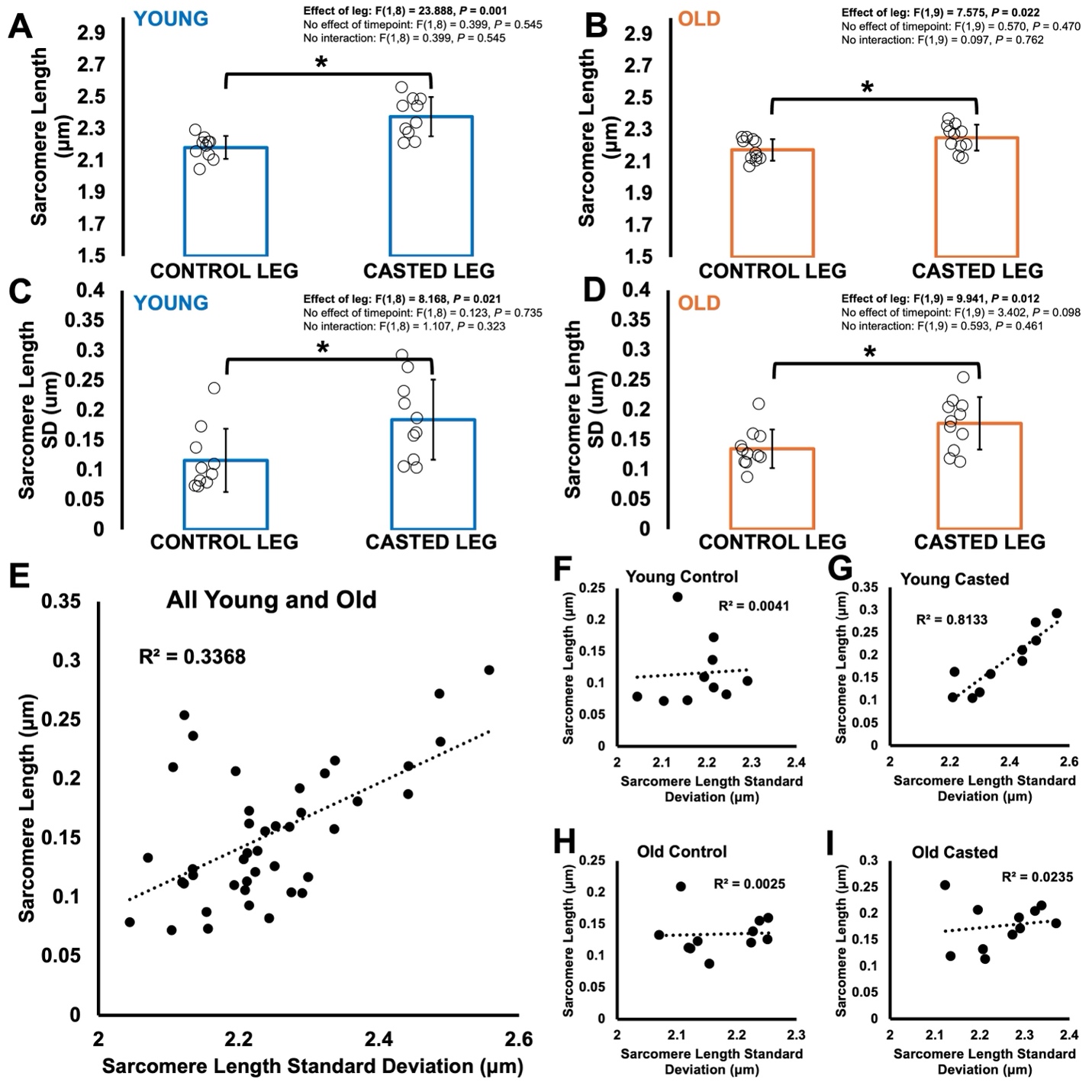
**

**Supplemental Figure S1:** Differences in sarcomere length (**A-B**) and sarcomere length standard deviation (SD) (estimate of sarcomere length non-uniformity) (**C-D**) between control and casted legs in young (n = 10) and old (n = 11) rats, with post-cast and 4 wk recovery time points combined because there were effects of leg but not time. Data are displayed as mean ± standard deviation. *Difference between indicated points (P < 0.05).

**Supplemental Figure S2: Ratio of fascicle length (FL) measured using ultrasound to FL measured on dissected fascicles from the same muscles**

|  |  | **Ultrasound FL (mm)** | **Dissected FL (mm)** | $\frac{\boldsymbol{Ultrasound FL}}{\boldsymbol{Dissected FL}}$ |
| --- | --- | --- | --- | --- |
| **Young** | **Post-cast** | 10.44 | 10.35 | 1.01 |
|  | **4 wk recovery** | 13.43 | 12.66 | 1.06 |
| **Old** | **Post-cast** | 9.14 | 8.71 | 1.05 |
|  | **4 wk recovery** | 11.16 | 10.70 | 1.04 |
| **Average** |  |  |  | 1.04 |
